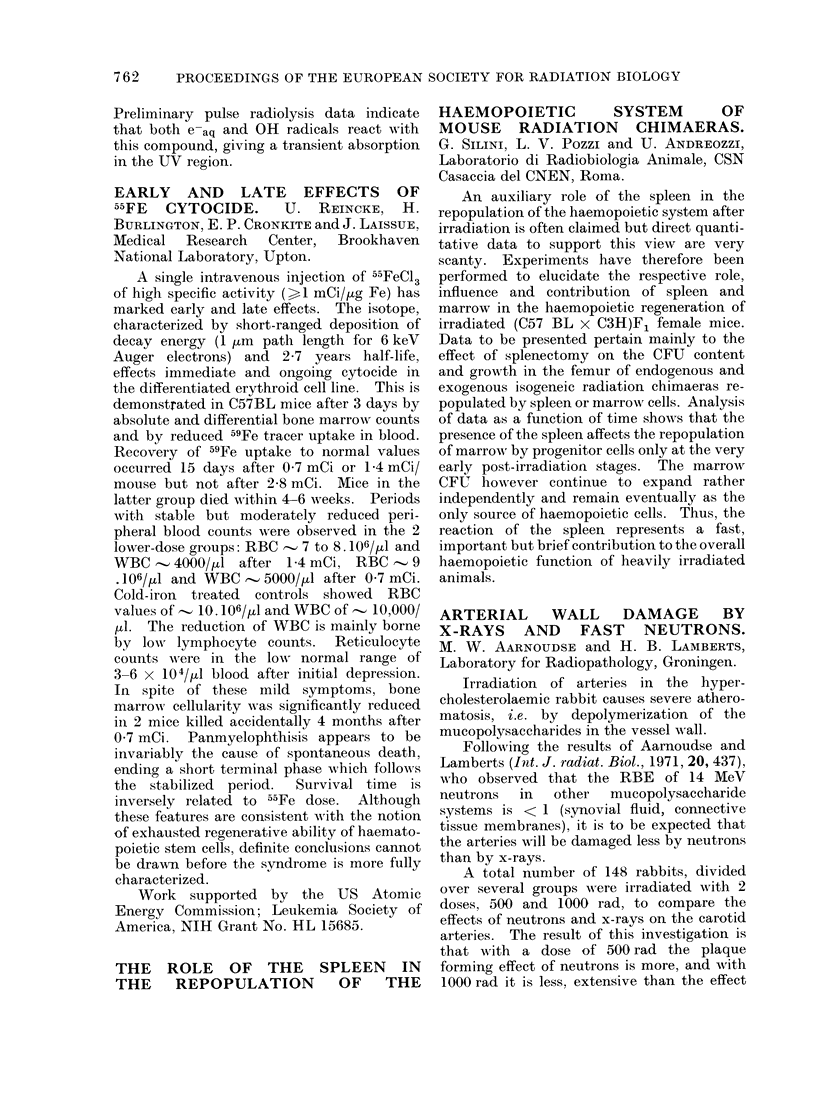# Proceedings: Early and late effects of 55Fe cytocide.

**DOI:** 10.1038/bjc.1975.325

**Published:** 1975-12

**Authors:** H. Burlington, E. P. Cronkite, J. Laissue


### EARLY AND LATE EFFECTS OF $^{55}$FE CYTOCIDE.

U. Reincke, H. Burlington, E. P. Cronkite and J. Laissue, Medical Research Center, Brookhaven National Laboratory, Upton.

A single intravenous injection of $^{55}FeCl_3$ of high specific activity ($\geqslant$1 mCi/$\mu$g Fe) has marked early and late effects. The isotope, characterized by short-ranged deposition of decay energy (1 $\mu$m path length for 6 keV Auger electrons) and 2·7 years half-life, effects immediate and ongoing cytocide in the differentiated erythroid cell line. This is demonstrated in C57BL mice after 3 days by absolute and differential bone marrow counts and by reduced $^{59}$Fe tracer uptake in blood. Recovery of $^{59}$Fe uptake to normal values occurred 15 days after 0·7 mCi or 1·4 mCi/mouse but not after 2·8 mCi. Mice in the latter group died within 4–6 weeks. Periods with stable but moderately reduced peripheral blood counts were observed in the 2 lower-dose groups: RBC $\sim$ 7 to 8.10$^6$/$\mu$l and WBC $\sim$ 4000/$\mu$l after 1·4 mCi, RBC $\sim$ 9 .10$^6$/$\mu$l and WBC $\sim$ 5000/$\mu$l after 0·7 mCi. Cold-iron treated controls showed RBC values of $\sim$ 10.10$^6$/$\mu$l and WBC of $\sim$ 10,000/$\mu$l. The reduction of WBC is mainly borne by low lymphocyte counts. Reticulocyte counts were in the low normal range of 3–6 $\times$ 10$^4$/$\mu$l blood after initial depression. In spite of these mild symptoms, bone marrow cellularity was significantly reduced in 2 mice killed accidentally 4 months after 0·7 mCi. Panmyelophthisis appears to be invariably the cause of spontaneous death, ending a short terminal phase which follows the stabilized period. Survival time is inversely related to $^{55}$Fe dose. Although these features are consistent with the notion of exhausted regenerative ability of haematopoietic stem cells, definite conclusions cannot be drawn before the syndrome is more fully characterized.

Work supported by the US Atomic Energy Commission; Leukemia Society of America, NIH Grant No. HL 15685.